# Dual induction of caspase 3- and transglutaminase-dependent apoptosis by acyclic retinoid in hepatocellular carcinoma cells

**DOI:** 10.1186/1476-4598-10-4

**Published:** 2011-01-09

**Authors:** Hideki Tatsukawa, Tetsuro Sano, Yayoi Fukaya, Naoto Ishibashi, Makiko Watanabe, Masataka Okuno, Hisataka Moriwaki, Soichi Kojima

**Affiliations:** 1 Molecular Ligand Biology Research Team, Chemical Genomics Research Group, Chemical Biology Department, RIKEN Advanced Science Institute, Wako, Saitama 351-0198, Japan; 2 Pharmaceutical Development, Pharmaceutical Division, KOWA Company, Ltd., Chuo, Tokyo 103-8433, Japan; 3 Tokyo New Drug Research Laboratories, Pharmaceutical Division, KOWA Company, Ltd., Higashimurayama, Tokyo 189-0022, Japan; 4 Department of Gastroenterology, Gifu University School of Medicine, Gifu 501-1194, Japan

## Abstract

**Background:**

Hepatocellular carcinoma has a high mortality rate due to its rate of recurrence. Acyclic retinoid prevents recurrence of hepatocellular carcinoma in patients after surgical removal of their primary tumors by inducing apoptosis in hepatocellular carcinoma cells, although the molecular mechanisms of action are not understood.

**Methods:**

Human hepatocellular carcinoma cells in culture, as well as nude mice transplanted with hepatocellular carcinoma cells and rats given with *N*-diethylnitrosamine were treated with acyclic retinoid. Changes in activated caspase 3 and transglutaminase 2 (TG2) levels, Sp1 cross-linking and its activities, expression of epidermal growth factor receptor, and apoptotic levels were measured.

**Results:**

Acyclic retinoid simultaneously stimulated the activation of caspase 3, and the expression, nuclear localization and crosslinking activity of TG2, resulting in crosslinking and inactivation of the transcription factor, Sp1, thereby reducing expression of epidermal growth factor receptor and cell death in three hepatocellular carcinoma cell lines. These effects were partially restored by a caspase inhibitor, transfection of antisense TG2, restoration of functional Sp1, or an excess of epidermal growth factor. Nuclear expression of TG2 and crosslinked Sp1, as also activated caspase 3 were found in both hepatocellular carcinoma cells transplanted into nude mice and cancerous regions within the liver in *N*-diethylnitrosamine-induced hepatocarcinogenesis model in rats, following treatment of animals with acyclic retinoid.

**Conclusions:**

Treatment with acyclic retinoid produces a dual activation of caspase 3 and TG2 induced apoptosis of hepatocellular carcinoma cells via modification and inactivation of Sp1, resulting in reduced expression of epidermal growth factor receptor.

## Background

Hepatocellular carcinoma (HCC) has high mortality rate because of it frequent rate of recurrence [1]. Acyclic retinoid (ACR), a synthetic retinoid, prevents the recurrence and development of HCC in patients after surgical removal of the primary tumors by inducing apoptosis in HCC cells [2,3]. Retinoid X receptor (RXR) α is highly phosphorylated and loses its activity as a transcriptional factor during carcinogenesis in HCC [4]. ACR prevents this aberrant hyper-phosphorylation of RXRα by suppressing the Ras-extracellular signal regulated kinase (Erk) pathway, thereby restoring RXRα's activity in response to physiological concentrations of 9-*cis *retinoic acid (9-*cis *RA) [5]. We therefore proposed that this restoration of RXRα transcriptional activity is a basis for ACR's activity to control aberrant cell growth and induce apoptosis. However, the possibility that genes under the control of RARα/RXRv are upregulated by ACR, thereby mediating ACR's effect in suppressing aberrant growth and/or inducing apoptosis, has not been fully elucidated. ACR downregulates epidermal growth factor receptor (EGFR) signals due to suppression of transforming growth factor (TGF) α in both HCC cells and human squamous cell carcinoma cells undergoing apoptosis [6,7]. ACR induces the expression of interferon receptor, and also the expression and activity of signal transducer and activator of transcription (STAT) 1 during suppression of cell growth and induction of HCC cell apoptosis [8]. However, it is unclear whether these phenomena are dependent on the restoration of RARα/RXRα.

Transglutaminase 2 (TG2) is a member of a family of crosslinking enzymes that catalyze a post-translational modification of proteins by a calcium-dependent crosslinking reaction that forms N-ε (γ-glutamyl) lysine bonds [9-12]. TG2 has been implicated in apoptosis, although the mechanisms are unknown. Recently, we demonstrated that TG2 induces caspase-independent apoptosis in ethanol-treated hepatocytes by crosslinking and inactivation of the general transcription factor, Sp1, thereby reducing Sp1-dependent expression of growth factor receptors [9,13]. However, whether TG2-induced apoptosis pathway is involved in apoptotic signaling in other cell types or is induced by stimulation with anti-cancer reagents remains unclear.

Piedrafita *et al. *[14] reported that retinoid-induced apoptosis of T cells accompanies degradation of Sp1 downstream of the caspase pathway. Shao *et al. *[15] found that ACR inhibits the growth of HCC cells by reducing the expression of an Sp1-transactiviable gene, fibroblast growth factor receptor 3 (FGFR3) [16].

These reports suggest that Sp1 and/or its regulating genes are important in ACR-induced apoptosis pathway in HCC cells. We have therefore tested the hypothesis that ACR can restore the expression of TG2 by preventing phospho-inactivation of RXRα, and downregulate the expression of growth factor receptors by inactivating Sp1 due to both caspase-dependent degradation and TG2-dependent crosslinking. We have used HCC cells in culture and *in vivo *models of both transplantation of HCC into nude mice and *N*-diethylnitrosamine (DEN)-induced rat hepatocarcinogenesis.

## Methods

### Materials

ACR (NIK-333) was supplied from Kowa Company, Ltd. (Tokyo, Japan). Anti-TG2 monoclonal antibody (TGase II, Ab-1) was purchased from NeoMarkers (Fremont, CA). Anti-TG2 polyclonal antibody was produced as previously described [13]. Mouse anti-Sp1 (IC6), rabbit anti-Sp1 (PEP2), anti-EGFR, anti-c-Met, anti-FGFR1 antibodies were bought from Santa Cruz Biotechnology (Santa Cruz, CA).

Mouse anti-GAPDH antibody was from Millipore (Billerica, MA). Anti-Bcl-X_L _and anti-cleaved caspase 3 antibodies were from Cell Signaling Technology (Danvers, MA). Horseradish peroxidase (HRP)-conjugated goat anti-rabbit or mouse IgG was from Jackson ImmunoResearch Laboratories (West Grove, PA). Viable cells were measured using a cell counting kit-8 (Dojindo; Tokyo, Japan). 5-(biotinamido) pentylamine, a biotinylated primary amine substrate for TG2 was provided by Pierce Biotechnology (Rockford, IL). A caspase-3 specific inhibitor, zDEVD-fmk, and Hoechst 33258 came from Calbiochem-Novabiochem (La Jolla, CA). Anti-crosslinked Sp1 (CLSp1) antibody was made in rabbits, and purified as previously described [13].

### Cells and plasmids

A HCC cell line, JHH-7 cells kindly supplied by Dr. Matsuura (Jikei University School of Medicine, Tokyo, Japan) [17] were maintained in ASF104 medium (Ajinomoto, Tokyo, Japan). HC cells, a normal human hepatocyte cell line purchased from Cell Systems (Kirkland, WA), were cultured in CS-C complete medium (Kirkland, WA) [4]. HuH-7, HepG2, and HelaS3 cells were maintained in RPMI 1640 medium containing 10% FBS. The expression vector for human Sp1 (*Sp1-pCIneo*) was constructed as previously described [18]. The TG2, Sp1, and EGFR siRNA-expressing lentiviral vectors were constructed in the pSIH-H1 shRNA vector (SBI System Biosciences, CA). A GC3-Luc vector, containing 3 sequential repeats of GC box motifs derived from the *EGFR *promoter [19] and its TATA box sequence upstream of the luciferase cDNA, was generated by inserting a synthesized oligodeoxynucleotide cassette into the pGL3 vector (Promega Corp., WI).

### Transient transfection

Transfections and assays of luciferase activity were performed with a combination of UNIFECTOR lipofection reagent (B-Bridge International, Inc.; Mountain View, CA) and luciferase reporter genes (firefly- and *Renilla*-Luc) as previously described [20], with further details being provided in the Additional file 1.

### TG2 knockdown

Knockdown of TG2 was performed by transfection of anti-sense (AS) or siRNA to TG2 in JHH-7 cells, suppressing the expression of TG2 protein ~50% and ~70%, respectively (Additional file 2 Figure S1)

### Preparation of whole lysates and nuclear extracts

Whole lysates were prepared in Hepes buffer containing 10 mM CHAPS and protease inhibitors. Nuclear extracts were prepared as previously described [20].

### Western blotting

Western blotting was carried out as previously described [20], using combinations of 1 μg/ml each of anti-Sp1, anti-CLSp1, or anti-TG2 antibody and HRP-conjugated goat anti-rabbit/mouse IgG (1:1,000 dilution). Reactants were detected with Enhanced Chemiluminescence reagents (GE Healthcare, Buckinghamshire, UK).

### Reverse transcriptase-polymerase chain reaction (RT-PCR)

RT-PCR was done as before [18], using sets of specific primers summarized in Additional file 3 Table S1.

### Staining of cells

Cells grown on cover slips were fixed with 10% formalin in culture medium. They were permeabilized with 0.3% Triton X-100 in TBS (pH 7.4), and stained with the antibodies given in each figure legend. Apoptosis was detected by the terminal deoxynucleotidyl transferase-mediated dUTP nick end-labeling (TUNEL) method with the *In Situ *Cell Death Detection Kit (Roche Diagnostics GmbH; Mannheim, Germany). Digital images of cells were obtained by confocal microscope (Carl Zeiss, Inc. Germany), and digital images recorded.

### Animal experiments

One week after JHH-7 cells (2 × 10^6 ^/50 μl) had been transplanted into the spleens of nude mice aged 6 weeks (Balb/c Slc-nu/nu, Japan SLC Inc., Shizuoka, Japan), ACR (100 mg/kg/day) or vehicle (soybean oil) was administrated by gavage at 10 μl/g body weight once a day on consecutive days for 3 weeks. The DEN-induced rat hepatocarcinogenesis model was used as previously described [21]. Briefly, 6-week old rats (F344/N SLC; Japan SLC Inc., Shizuoka, Japan) were given drinking water containing 40 ppm DEN (Tokyo Kasei Kogyo Co., Tokyo, Japan) for 15 weeks to produce liver neoplasms. ACR (40 and 80 mg/kg) or vehicle (soybean oil) was administered orally with a stomach tube at 5 μl/g body weight for 14 weeks. Experiments were performed in accordance with protocols approved by the RIKEN Institutional Animal Use and the Care Administrative Advisory Committees.

### Immunohistochemistry

Immunohistochemistry were performed as before [13]. Livers were removed, fixed in 10% formalin, and embedded in paraffin wax. Sections were prepared and stained with anti-CLSp1, anti-TG2, anti-cleaved caspase 3, and anti-EGFR polyclonal antibodies. Staining signals were enhanced using an ABC kit (VECTASTAIN) and developed with DAB substrate.

### Statistical analysis

Quantitative data are given as means ± SD. Student's *t *test was used to evaluate differences between 2 groups. In comparing data from the vehicle group with those from groups treated with ACR at doses of 25, 50, and 100 mg/kg body weight, the level of serum AFP and the number of AFP-positive mice were analyzed by Dunnett's multiple comparison and Fisher's exact probability test, respectively. A *p*-value of <0.05 was considered statistically significant.

## Results

### ACR induces both caspase- and TG2-dependent apoptosis pathways

**Figure 1:**
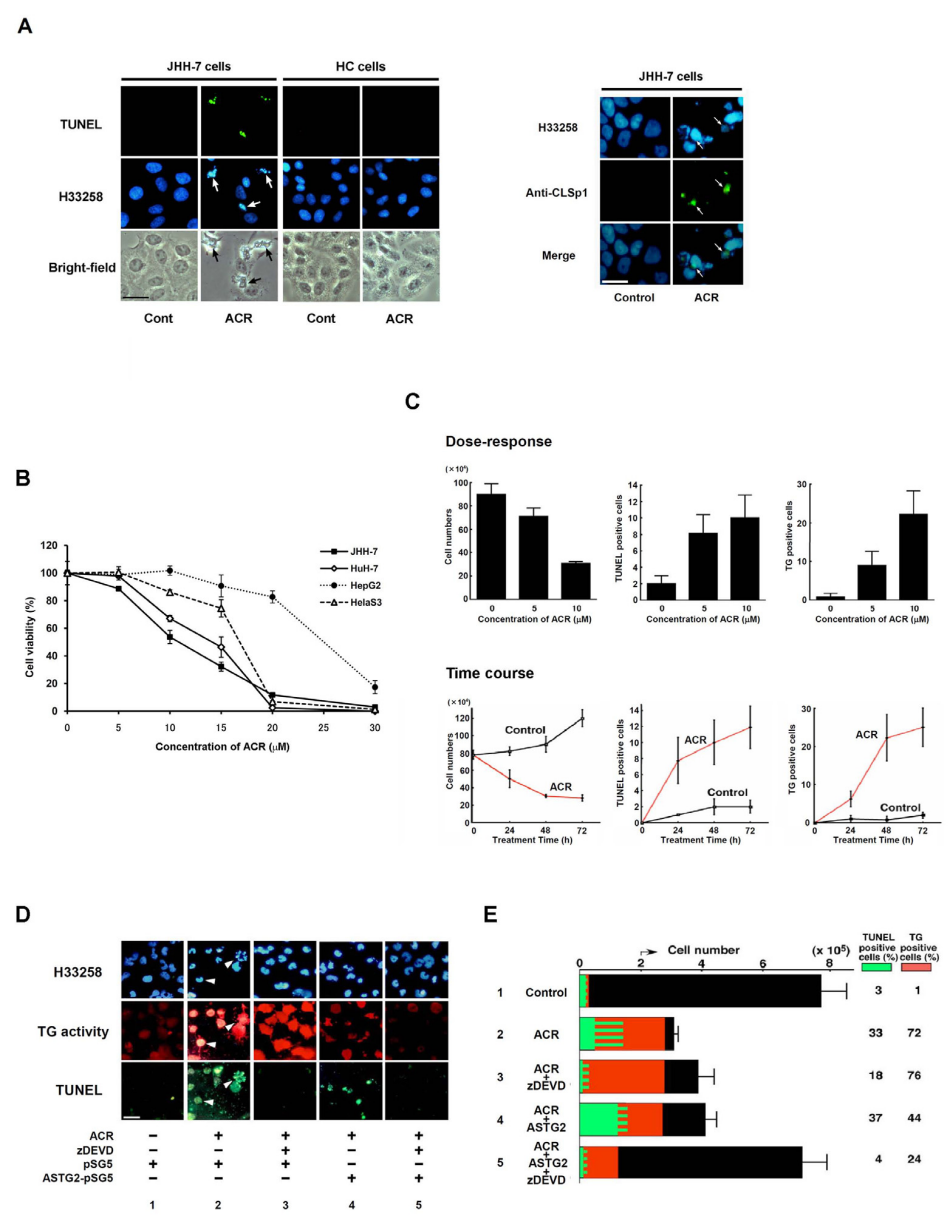
**Induction of caspase 3 and TG2- dependent apoptosis by ACR in JHH-7 cell cultures**. *A*, JHH-7 and HC cells were seeded in 35 mm dishes containing glass coverslips at 2 × 10^5^/dish, and treated with 10 μM ACR or vehicle (0.1% ethanol) for 24 h. Cells were fixed and stained combination with Hoechst 33258, and TUNEL (*left panels*) or anti-CLSp1 antibody (*right panel*). Scale bar, 50 μm. *B*, JHH-7, HuH-7, HepG2 and HelaS3 were seeded at 1 × 10^4 ^cells/96 well microplates and treated with the indicated concentrations of ACR or vehicle for 24 h. Viable cell counts are plotted as percentages of each control culture treated with vehicle. *C*, JHH-7 cells were seeded as before and treated either with the indicated concentrations of ACR for 48 h, or with 10 μM ACR for the indicated times. Cells were fixed and stained with Hoechst 33258, TUNEL, and anti-TG2 antibody. The numbers of total and apoptotic cells with TUNEL or TG2 positivity in each dish were counted and plotted. *D*, JHH-7 cells were seeded as before and transfected with either pSG5 or anti-sense (AS) TG2-pSG5. The next day cells were treated with either vehicle or 10 μM ACR for 24 h in the presence or absence of 100 μM zDEVD-fmk, with 1 mM 5-(biotinamido)-pentylamine being included during the last 2 h incubation. Cells were fixed and stained with Hoechst 33258 (*upper panels*), TRITC-conjugated streptavidin (*middle panels*), and TUNEL (*bottom panels*). Arrowheads indicate apoptotic cells with chromatin condensation. Scale bar, 50 μm. *E*, JHH-7 cells were treated as in (*C*). The numbers of total and apoptotic cells with TUNEL (green colors) or TG2 (orange colors) positivity in each dish were counted and plotted as bar graphs. Their percentages relative to total cell number are given on the right hand-side of each bar graph. Panels *A-E *show representative results from 3 different experiments with similar results.

ACR induced TUNEL-positive apoptosis of JHH-7 cells, but not normal hepatocyte HC cells (Figure 1A, *left panel*). Apoptotic JHH-7 cells were also positive for crosslinked Sp1 (CLSp1; Figure 1A, *right panel*). Strong immunofluorescent spots were obvious in cells undergoing severe apoptosis (Figure 1A, *right panel*, arrows). JHH-7 cells were the most sensitive to ACR of the 3 HCC cell lines (JHH-7, HuH-7, HepG2) and HelaS3 cells (Figure 1B). They showed similar TG2/TUNEL/CLSp1-positive apoptosis following ACR treatment (*data not shown*). Consistent with previous findings with another HCC cell line (HuH-7) [6], ACR treatment of JHH-7 cells, but not HC cells, suppressed phosphorylation of RXRα without affecting the expression of RXRα (Additional file 4 Figure S2A), prevented phospho-inactivation of RXRα, and enhanced the expression of TG2 (Additional file 4 Figure S2B).

Reciprocally in parallel with a dose- and time-dependent decrease in cell number (Figure 1C, *left panels*), both TUNEL (Figure 1C, *middle panels*) and TG2 positivity (Figure 1C, *right panels*) increased in ACR-treated JHH-7 cells undergoing apoptosis. ACR-induced apoptosis was partially blocked by either the inclusion of the caspase inhibitor, z-DEVD (Figure 1D and 1E, *sample 3*) or knocking down by 50% TG2 expression with anti-sense (AS) TG2 (Figure 1D and 1E, *sample 4*; Additional file 2 Figure S1A), whereas apoptosis was almost completely blocked by their combined inhibition (Figure 1D and 1E, *sample 5*). These results suggest that ACR-induced apoptosis is dependent on both caspase 3 and TG2 activation.

In ACR-treated JHH-7 cells ACR had markedly increased levels of CLSp1 (Figure 1A, *right panel *and Additional file 5 Figure S3A, *lane 4*), whereas levels of the Sp1 monomer decreased (Additional file 5 Figure S3A, *lane 2*), thereby reducing its DNA binding activity (Additional file 5 Figure S3B) and transactivation activity (Additional file 5 Figure S3C), as previously seen in ethanol-induced hepatocyte apoptosis [13]. Impaired Sp1 activity was partially improved either by inhibition of caspase or TG2 knockdown by transfection of ASTG, and almost completely restored by their combination, as also by overexpression of Sp1. These results suggest that both caspase- and TG2-dependent pathways lead to silencing of Sp1 activity, which correlates with cell viability (Additional file 5 Figure S3D).

### Reduced expression of growth factor receptors as the major Sp1 transcriptional targets in ACR-treated JHH-7 cells undergoing apoptosis

**Figure 2:**
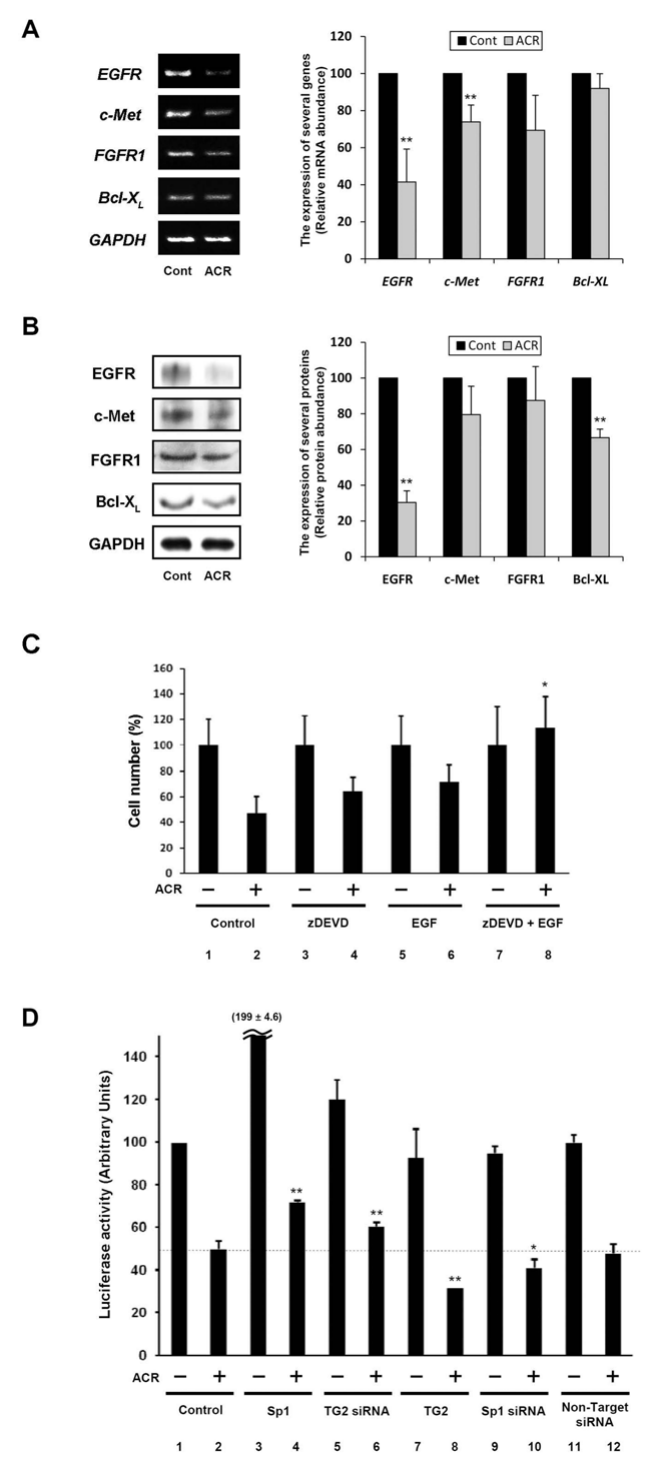
**Induction of caspase 3- and TG2-dependent apoptosis by ACR via a reduction in the expression of EGFR due to silencing of Sp1**. *A*, JHH-7 cells were treated with 10 μM ACR or vehicle for 12 h. Cells were harvested and mRNA expression of indicated genes was determined by RT-PCR. Bar graphs show densitometrically determined relative mRNA abundance normalized to *GAPDH *mRNA levels. **P < 0.01 compared to each control. *B*, JHH-7 cells were treated with 10 μM ACR or vehicle for 24 h. Cells were harvested and protein expression of indicated proteins was determined by Western blotting. The bar graph shows densitometrically determined relative protein abundance normalized to GAPDH protein levels. **P < 0.01 compared to each control. *C*, JHH-7 cells were treated with 10 μM ACR for 24 h in the presence or absence of 100 μM zDEVD, 50 ng/ml EGF or a combination of the two, and the numbers of viable cells were determined after trypsinization by Trypan Blue exclusion. Results shown are means ± SD (n = 3). *P < 0.05, compared to ACR-treated sample from control cells (*lane 2*). *D*, One day after transfection of JHH-7 cells with *EGFR *promoter GC3-Luc (1 μg/dish), cells were treated with 10 μM ACR for 24 h, co-transfected with Sp1 (*lanes 3 and 4*), TG2 shRNA (*lanes 5 and 6*), TG2 (*lanes 7 and 8*), Sp1 shRNA (*lanes 9 and 10*), and non-target siRNA (*lanes 11 and 12*) expression vector, and cell lysates were prepared. Luciferase activity of each cell lysate was determined. Results shown are means ± SD (n = 3). *P < 0.05, **P < 0.01 compared to ACR-treated control sample from control cells (*lane 2*). *Panels A-D *show representative results from three different experiments with similar results.

ACR-treated JHH-7 cells expressed decreased levels of EGFR at both mRNA (Figure 2A; 2.5-fold reduction in quantitative PCR) and protein (Figure 2B) levels. Although protein levels of c-Met and FGFR1 remained largely unaltered, mRNA levels of c-Met and FGFR1 decreased slightly following ACR-treatment. mRNA of Bcl-X_L _was unchanged, but moderately altered at the protein level. ACR induced activation of caspase 3, but not its expression (Additional file 6 Figure S4A and S4B, *respectively*). While a single treatment with either a caspase inhibitor, z-DEVD (Figure 2C, *lane 4*) or overloading EGF (Figure 2C, *lane 6*) partially prevented a reduction in cell number in ACR-treated JHH-7 cells, combined treatment completely prevented this reduction (Figure 2C, *lane 8*).

**Figure 3:**
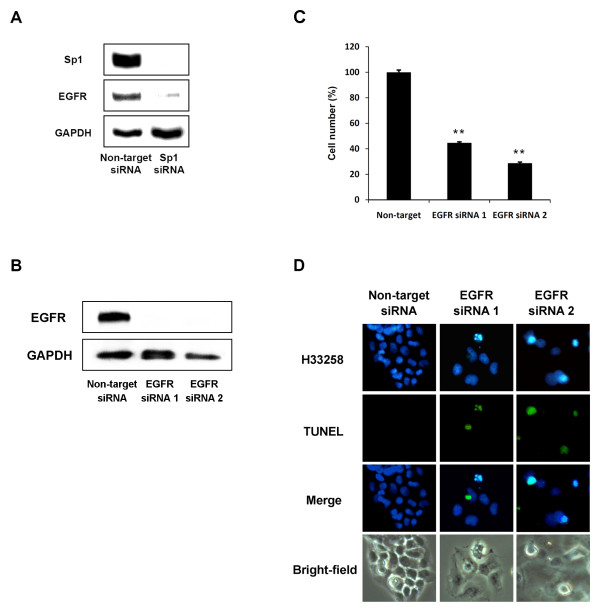
**Induction of apoptosis in JHH-7 cells using Sp1 and EGFR siRNAs**. *A*, JHH-7 cells overexpressing non-target or Sp1 siRNA were harvested and protein levels of Sp1, EGFR, and GAPDH determined by Western blotting. *B*, JHH-7 cells overexpressing non-target or 2 kinds of EGFR siRNAs were harvested and protein levels of EGFR and GAPDH determined by Western blotting. *C*, Numbers of viable cells were counted 2 days after seeding these cells on 35 mm dishes. Results shown are the number of viable cells relative to the controls, expressed as % ± S.D. **P < 0.01 compared to control cells. *D*, The seeded cells on cover slips in 35 mm dishes were fixed and stained with Hoechst (*upper panels*) and TUNEL (*second panels*). *Panels A-D *show representative results from 3 different experiments with similar results.

To determine whether reduced expression of EGFR was due to Sp1 inactivation, transactivation of a chimeric reporter gene-construct in which expression was driven by 3 tandem functional GC box motifs derived from the EGFR promoter was monitored. ACR-treatment decreased the transactivational activity of the EGFR gene promoter (compare Figure 2D, *lanes 1 and 2*), which was partially prevented by overexpressing Sp1 (compare Figure 2D, *lanes 2 and 4*) or downregulating TG2 expression by 70% (compare Figure 2D, *lanes 2 and 6*; Additional file 2 Figure S1B). It was partially reversed by overexpression of TG2 (compare Figure 2D, *lanes 2 and 8*) and Sp1 inactivation with siRNA (compare Figure 2D, *lanes 2 and 10*). Sp1 inactivation with siRNA also reduced expression of EGFR protein (Figure 3A). In hepatocytes, treatment with Sp1 siRNA had previously decreased cell viability ([13]; *data not shown here*). siRNA knockdown of EGFR led to apoptosis (Figure 3B-3D). These results suggest that transcriptional reduction of *EGFR *due to a reduction in Sp1 activity may partially explain ACR-induced apoptosis of HCC cells.

### ACR suppresses both transplant of human HCC cells in nude mice and DEN-induced rat hepatocarcinogenesis by inducing apoptosis accompanying the emergence of nuclear TG2 and CLSp1

**Figure 4:**
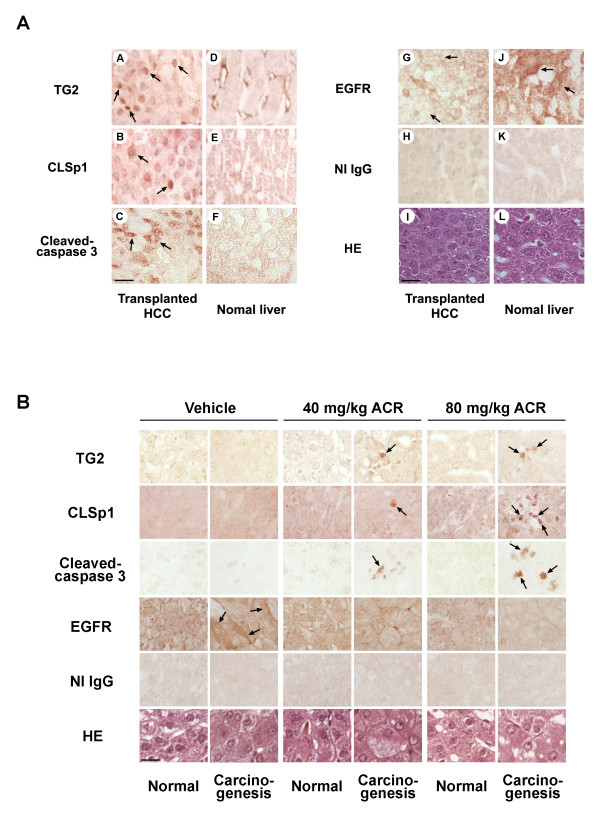
**Nuclear accumulation of TG2 and CLSp1 observed in the liver of nude mice transplanted with JHH-7 cells, and in DEN-treated rats with liver cancer after ACR treatment**. *A*, Liver sections including normal (*panels D-F and J-L*) and metastatic areas (*panels A-C and G-I*) from JHH-transplanted nude mice following treatment with ACR were stained with polyclonal anti-TG2 (30 μg/ml; *panels A, D*), anti-CLSp1 (30 μg/ml; *panels B, E*), anti-cleaved caspase 3 (10 μg/ml; *panels C, F*), anti-EGFR (10 μg/ml; *panels G, J*), and non-immune antibodies (NI IgG; 30 μg/ml; *panels H, K*). *B*, Liver sections from normal and neoplastic areas in DEN-treated rats following treatment with vehicle or ACR (at 40 and 80 mg/kg) were stained as in Figure 4A. The signals were enhanced with an ABC kit and developed with DAB substrate. Sections were counterstained with hematoxylin-eosin (HE; Figure 4A, *panels I, L*, and Figure 4B, *bottom panels*). Arrows indicate signals under the levels for each antigen. Scale bar, 50 μm.

Finally, the *in vivo *effect of ACR was examined in the 2 animal models. Using the transplant model in mice, where ACR dose-dependent reduction of serum levels of a tumor marker for HCC, α-fetoprotein (AFP) and the incidence of HCC (Additional file 7 Table S2), nuclear TG2 and CLSp1 increased in cancerous liver cells of ACR-treated nude mice transplanted with the JHH-7 cell line (Figure 4A, *panels A and B, respectively*) compared with adjacent normal liver (Figure 4A, *panels D and E*). Significant induction of TG2 and activation of caspase 3 occurred in metastatic areas in nude mice transplanted with JHH-7 cells after treatment with ACR (Figure 4A, *panels A and C, respectively*). Moreover, EGFR levels in the metastatic areas were lower than in normal areas of the same liver (compare Figure 4A, *panels G and J*). Similar results were obtained in the rat model of DEN-induced hepatocarcinogenesis, in which ACR's anti-cancer effect has been reported [21]. Simultaneous induction of TG2, CLSp1, and activation of caspase 3 occurred in paralleled with a reduction in EGFR (Figure 4B).

## Discussion

**Figure 5:**
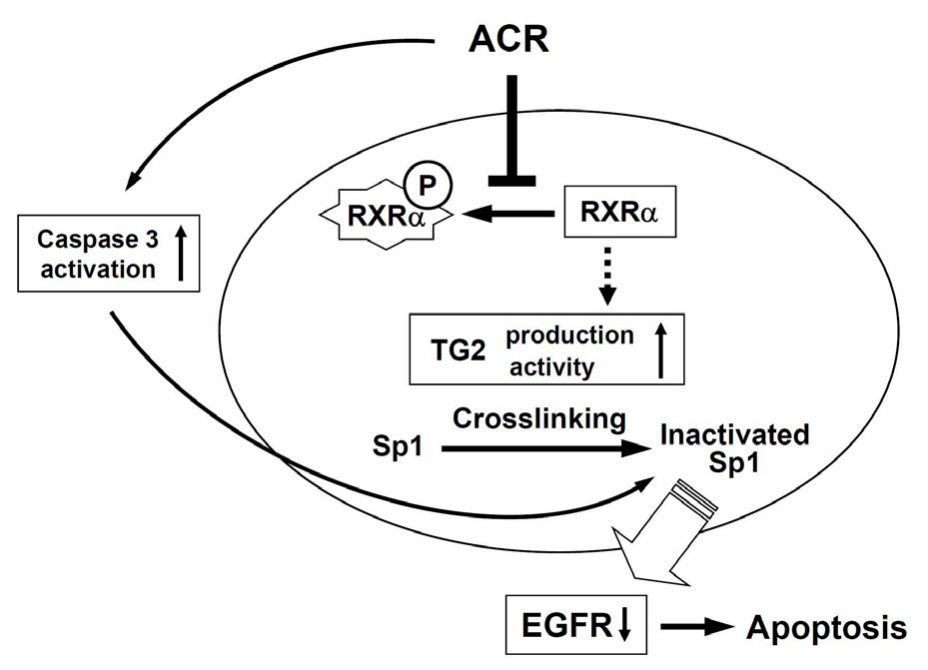
**Schematic diagram showing the molecular mechanism by which ACR causes HCC apoptosis by restorating phospho-inactivated RXRα followed by enhanced TG2-mediated crosslinking/silencing of Sp1, thereby reducing EGFR-medicated EGF signaling**.

The data show that: (i) ACR suppresses the hyper-phosphorylation of RXRα, restored its transcriptional function, and enhanced the expression of TG2 and its nuclear accumulation, along with caspase 3 activation; (ii) Sp1 is crosslinked by TG2 and degraded by caspase 3, resulting in loss of its activity; and (iii) expression of Sp1-regulated target genes, such as EGFR (critical for cell survival), decrease, culminating in apoptosis of the cancer cells (Figure 5). The results of *in vitro *findings were confirmed by the *in vivo *models of nude mice transplanted with JHH-7 cells and DEN-induced hepatocarcinogenesis in rats (Figure 4). The recurrence of HCC in these animal models remains to be elucidated.

ACR treatment induced apoptosis in HCC cells (JHH-7 and HuH-7), but not in normal hepatocyte cells (HC cells) (Figure 1A and 1B). As a clue to a reason for the difference, we found that both expression and phosphorylation levels of RXRα were much higher in HCC cells than in HC cells, and that ACR suppressed its phosphorylation levels without altering its expression level (Additional file 4 Figure S2A), as previously shown [5]. In further previous work, we had demonstrated that 2 amino acids in RXRα, T82 and S260, were phosphorylated in HCC, but not in HC cells [4]. Therefore, phosphorylation of RXRα observed in JHH-7 cells was referred to as "hyperphosphorylation". However, RARα and RARβ were phosphorylated not only in JHH-7 cells, but also in HC cells, and ACR downregulated their phosphorylation in both cases (Additional file 4 Figure S2A). Phosphorylation was not detected in the other 3 subtypes of RXR and RAR (Additional file 4 Figure S2A). Therefore, phosphorylation of RXRα was only specific in cancer cells, which could be a reason for the selective apoptosis of cancer cells by ACR treatment.

It is noteworthy that treatment with either antisense of TG2 or inhibitors of caspase 3 only partially blocked ACR-induced apoptosis, whereas their simultaneous inhibition completely prevented apoptosis, suggesting that TG2 and caspase 3 contribute independently to the induction of apoptosis (Figure 1D and 1E). We measured the activity of caspase 3 and TG2 in the presence of an inhibitor of each other's enzyme, such as zDEVD and cystamine. When cystamine suppressed ~50% of ACR induction in TG2 activity (compare the differences between *lanes 1 and 4 *with those between *lanes 2 and 5 *in Additional file 6 Figure S4D), it suppressed 60% of ACR induction in caspase 3 activity (compare the differences between *lanes 1 and 4 *with those between *lanes 2 and 5 *in Additional file 6 Figure S4C). On the other hand, when zDEVD completely suppressed ACR-induced increase in caspase 3 activity (compare the differences between *lanes 1 and 4 *with those between *lanes 3 and 6 *in Additional file 6 Figure S4C), 50% of an increase in the TG2 activity remained (compare the differences with *lanes 1 and 4 *with those between *lanes 3 and 6 *in Additional file 6 Figure S4D). The data suggest that TG2 and caspase 3 influenced each other with a higher hierarchy of TG2 over caspase 3 in the contribution to the apoptosis of HCC induced by ACR. Synergism between inhibition in caspase and overloading of EGF in preventing apoptosis also suggests that both the caspase 3- and EGFR-dependent pathways exist (Figure 2C).

Expression of EGFR is regulated by Sp1 [19,22], and inhibition of EGFR signaling leads to growth inhibition, apoptosis, and cell cycle arrest of HCC cells [23,24]. We have linked these findings by showing that the downregulation of EGFR with siRNA induces apoptosis (Figure 3B-D), suggesting that inhibiting EGFR signaling via silencing Sp1 is a promising treatment strategy against HCC.

Induction of CLSp1 and the subsequent reduction in EGFR has been reproduced in ACR-treated HuH-7 cells (*data not shown*). In contrast, although Shao *et al. *[15] reported that ACR inhibits the cell growth through downregulation of FGFR3 expression and FGF-mediated signaling in HepG2 cells, this was not found to be the case in our ACR-treated JHH-7 cells (*data not shown*). These findings suggest that HCC cell lines differ in the way that growth factor receptors are involved in survival.

Whereas TG2 may be a substrate of caspase 3 during apoptosis of thymocytes, resulting in loss of transamidating function [25], TG2 in turn inhibits of apoptosis due to crosslinking and inactivation of caspase 3 in thapsigargin-mediated apoptosis of colon carcinoma cells [26]. In the latter article, thapsigargin treatment generated 2 additional biologically inactive species of caspase 3, viz. p40 and p64, via TG2-mediated crosslinking of caspase 3, thereby protecting cells from apoptosis. However, we failed to detect either p40 or p64 in our ACR-treated JHH-7 cells. We speculate that crosslinking of caspase 3 would be induced specifically by treatment with thapsigargin. Our data clearly shows that both caspase 3 and TG2 are functional in ACR-treated HCC cells, without apparent alteration of caspase 3 expression (Additional file 6 Figure S4A and 4B). These controversial results might be ascribed to differences in cell types and the nature of the apoptotic stimuli, although the precise mechanisms need to be elucidated.

Piedrafita and Pfahl [14] reported that caspase 3 directly cleaved and inactivated Sp1 in retinoid-treated T cells undergoing apoptosis. They showed that cleavages of PARP and Sp1 were simultaneously induced by caspase 3 and prevented with caspase inhibitors (zVAD-fmk and zDEVD-fmk). We anticipated that CLSp1 might also be partially cleaved by caspase 3; however, as molecular size differences would be too small to be recognized on the gel against a high molecular weight of CLSp1 detected at the top of the gel, we found no band shifts due to the cleavage. Hence, the possibility of simultaneous crosslinking and cleavage of Sp1 by TG2 and caspase 3, respectively, cannot be ruled out, even though we saw no truncated Sp1 with a Mw of 68 kD in ACR-treated HCC cells.

ACR-treated JHH show enhanced nuclear localization of TG2; nuclear localization of TG2 is also important for induction of TG2-dependent apoptosis. Peng *et al. *[27] reported that TG2 binds importin-α3, an important factor in nuclear translocation, and therefore we are investigating the detail mechanism of TG2 nuclear localization accompanying ACR-induced apoptosis.

## Conclusions

Our new findings indicate that ACR induces both activation of caspase 3 as well as the expression and activation of TG2, which together initiate the apoptotic pathway via degrading/crosslinking and inactivation of the transcription factor, Sp1. Reduced expression of growth factor receptor genes (*e.g*. EGFR) also occurs. This dual activation of both caspase and TG-dependent apoptotic pathways could in part be central as mechanisms by which ACR inhibits tumor cell growth, resulting in the prevention of secondary tumors after treatment of primary HCCs (Figure 5). Future study should establish the possibility that regulation of TG2-dependent apoptotic pathway may help in the development of new therapies for the prevention of HCC.

## List of abbreviations

9-*cis *RA: 9-*cis *retinoic acid; ACR: acyclic retinoid; CLSp1: crosslinked Sp1; DEN: *N*-diethylnitrosamine; EGFR: epidermal growth factor receptor; FGFR3: fibroblast growth factor receptor 3; HCC: hepatocellular carcinoma; RXR: retinoid X receptor; TG2: transglutaminase 2.

## Competing interests

The authors declare that they have no competing interests.

## Authors' contributions

HT and TS performed the research, analyzed the data, and drafted the manuscript. YF helped with cell culture, transfection, immunostaining and Western blotting techniques. NI prepared the acyclic retinoid used in these studies. MW helped with immunostaining techniques. MO, HM and SK designed the research, interpreted the data, and revised the manuscript. All authors approved the final version of the manuscript.

## Supplementary Material

Additional file 1 **Additional text**. This text contains the additional "Methods" and "References" Click here for file

Additional file 2 **Figure S1: Efficiency of transfection with anti-sense and siRNA to TG2 in JHH-7 cells**. *A*, JHH-7 cells were seeded in 60 mm dishes at 6 × 10^5^/dish, and transfected with 4 μg of either empty vector (pSG5) or ASTG2-pSG5. Cells were harvested and the expression level of TG2 determined by Western blotting. Upper numbers in parentheses show the densitometrically determined relative protein abundance. *B*, JHH-7 cells were seeded in 60 mm dishes at 6 × 10^5^/dish, and transfected with 4 μg of vectors expressing either non-target siRNA or TG2 siRNA. Cells were harvested and the expression level of TG2 determined by Western blotting. Upper numbers in parentheses show the densitometrically determined relative protein abundance. *Panels A and B *show representative results from 3 different experiments with similar results. Click here for file

Additional file 3 **Table S1: Primers for RT-PCR and quantitative-PCR experiments**. The list of used specific primers for RT-PCR. Click here for file

Additional file 4 **Figure S2: ACR prevented phosphorylation and inactivation of RXRα, and stimulated the expression of TG2 in JHH-7 cells**. *A*, JHH-7 cells (*lane 1 and 2*) and HC cells (*lane 3 and 4*) were treated with 10 μM ACR or vehicle for 12 h. Cells were harvested and nuclear extracts were prepared. Phosphoproteins affinity-purified from each nuclear extract using the Phosphoprotein Purification Kit (QIAGEN) (*left panel*) as well as whole nuclear extracts (*right panel*), were subjected to SDS-PAGE, followed by Western blotting using the indicated antibodies against 6 different RXR/RAR or GAPDH. *B*, JHH-7 cells were transfected with either an empty vector (*columns 1-4*) or vectors expressing wild-type RXRα (*columns 5-8*), its alanine mutant T82A (unphosphorylated form; *columns 9-12*), or its aspartate mutant T82 D (phosphomimic; *columns 13-16*). The next day cells were treated either with 9-*cis *RA (9cRA; 6 μM) or its vehicle, or with and/or ACR (10 μM) for 24 h. Subsequently, levels of *TG2 *mRNA in cell lysates were quantified by RT-PCR (*upper panels*) and quantitative-PCR (*lower graphs*), where relative expression levels of TG2 were calculated in comparison with each control and then plotted. Treatment with 1 μM 9-cis-RA also gave basically similar results (data not shown), but the data obtained under treatment with 6 μM 9-cis-RA are shown here, giving the more significant differences. *Panels A and B *show representative results from 3 different experiments with similar results. Click here for file

Additional file 5 **Figure S3: Crosslinking and silencing of Sp1 in ACR-treated JHH-7 cell cultures undergoing apoptosis and its reversion by overexpression of Sp1**. *A*, JHH-7 cells were treated with 10 μM ACR for 24 h. The cells were harvested and nuclear extracts prepared. The levels of Sp1 and CLSp1 were assessed by Western blotting with an anti-Sp1 (*columns 1 and 2*) and CLSp1 (*columns 3 and 4*) antibodies, respectively. *B*, JHH-7 cells were transfected with 1.5 μg of either combination of *pCIneo*, *pSG5*, *Sp1-pCIneo*, or *anti-sense (AS) TG2-pSG5*. The next day they were treated with either 10 μM ACR or its vehicle in the presence or absence of 100 μM zDEVD-fmk for 24 h. Cells were harvested and nuclear extracts prepared. Sp1 DNA-binding activity of each nuclear extract (10 μg protein) was determined by gel-shift assay, using a consensus GC box as a probe (+cold; nuclear extracts + 50-fold excess of unlabeled probe, +anti-Sp1 IgG; nuclear extracts + 2 μg of anti-Sp1 antibody, +NI IgG; nuclear extracts + 2 μg of non-immune IgG). *C*, JHH-7 cells were transfected with 1.5 μg of a consensus GC3-Luc reporter and *Renilla*-Luc, plus a combination of *pCIneo*, *pSG5*, *Sp1-pCIneo *or *anti-sense (AS) TG2-pSG5*. The next day the cells were treated with 10 μM ACR for 24 h in the presence or absence of 100 μM zDEVD-fmk. Cell lysates were prepared and luciferase activity of each cell lysate determined. Results are means ± SD (n = 3). *D*, JHH-7 cells were transfected with either a combination of *pCIneo*, *pSG5*, *anti-sense (AS) TG2-pSG5*, *Sp1-pCIneo*, *Sp1 C domain-pCIneo*, Δ*C Sp1-pCIneo*. The next day the cells were treated with 10 μM ACR for 24 h. The number of viable cells was determined. Results are means ± SD (n = 4). *Panels A-D *show representative results from 3 different experiments with similar results. Click here for file

Additional file 6 **Figure S4: ACR stimulated activation of caspase 3 and TG2 in JHH-7 cells and the crosstalk between these proteins**. *A and B*, JHH-7 cells were treated with 10 μM ACR or the vehicle for 24 h. Cells were harvested and protein levels of activated caspase 3 and GAPDH determined by Western blots, using anti-cleaved-caspase 3 and anti-GAPDH antibodies (*A*); each of their mRNA expression was determined by RT-PCR (*B*). *C*, JHH-7 cells was seeded at 1 × 10^4 ^cells/96 well microplates and treated with 10 μM ACR or vehicle (0.1% ethanol) for 5 h in the presence or absence of either 100 μM zDEVD-fmk or 100 μM cystamine with 0.2 mM 5-(biotinamido)-pentylamine. Caspase 3 activity was measured using a Caspase-Glo 3/7 assay kit (Promega Corp., WI) as described in attached manual. Relative caspase 3 activity of each sample was calculated by normalization with the number of viable cells in the same sample measured with a cell counting kit-8 (Dojindo; Tokyo, Japan). *D*, JHH-7 cells seeded in 100 mm dishes at 1.6 × 10^6^/dish were treated as in (C). TG2 activity was measured as described in Additional file 1. Relative TG2 activity of each sample was calculated by normalization with the number of viable cells in the same sample, measured with a cell counting kit-8 (Dojindo; Tokyo, Japan). *Panels A-D *show representative results from 3 different experiments with similar results. Click here for file

Additional file 7 **Table S2: Suppression by ACR of metastasis and growth of human HCC cell line, JHH-7 cells transplanted into nude mice**. Nude mice that had been transplanted with JHH-7 were given orally with ACR with increasing concentrations (25, 50, and 100 mg/kg/day) as described detailed in the "Methods". Serum AFP was measured. Incidence was calculated based on level of the positive-AFP (more than 6 ng/ml). Cisplatin was used as a positive control. *p < 0.05 compared to control (Dunnett's multiple comparison test), #p < 0.05 compared to control (Fisher exact test).. Click here for file
