# Supplementary material for: Dual induction of caspase 3- and transglutaminase-dependent apoptosis by acyclic retinoid in hepatocellular carcinoma cells
Source: Mol Cancer. 2011 Jan 9;10:4. doi: 10.1186/1476-4598-10-4 (PMC3024303; doi:10.1186/1476-4598-10-4)
Supplement: Additional file 1 — Additional text. This text contains the additional "Methods" and "References" [file 1476-4598-10-4-S1.DOC]

Additional file 1, text

**Methods**

**Plasmids**

The expression vectors for a Sp1 fragment (*C terminal Sp1- pCIneo*) and a mutant Sp1 deficient in DNA binding domain (*∆C Sp1-pCIneo*) were also constructed by PCR, using primers, which amplified the DNA fragment including or lacking C-terminal domain of Sp1.

**Measurement of TG2 activity**

TG2 activity was measured as previously described [1]. Briefly, JHH-7 cells seeded in 100 mm dishes at 1.6×10^6^/dish were treated with 10 μM ACR or vehicle (0.1% ethanol) for 5 h in the presence or absence of either 100 μM zDEVD-fmk or 100 μM cystamine with 0.2 mM 5-(biotinamido)-pentylamine. Cells were harvested and lysed. Ten micrograms of homogenate protein was diluted to 50 μl with coating buffer (50 mM Tris-HCl, pH 7.4, 150 mM NaCl, 5 mM EGTA, 5 mM EDTA) and added to each well of a 96-well microplate, and the plate incubated overnight at 4 °C. Then 5% BSA, 0.01% SDS, 0.01% Tween 20 in borate-buffered saline (BBS; 100 mM boric acid, 20 mM sodium borate, 80 mM NaCl) was added to each well, and the incubation continued for 2 h at 37°C. Each well was rinsed once with 1% BSA, 0.01% Tween 20 in BBS. HRP-conjugated streptavidin (1:1000) in 1% BSA, 0.01% Tween 20 was added and incubated at room temperature for 1 h. The wells were rinsed 4 times with 1% BSA, 0.01% Tween 20 in BBS before the substrate solution was added to each well. After incubating at room temperature, the reactions were stopped by the addition of 1 N HCl to each well, and the presence of proteins into which 5-(biotinamido)-pentylamine had been incorporated was quantitated by measuring the absorbance at 450 nm in a microplate spectrophotometer (ARVO MX/Light; PerkinElmer, MA).

**Transient transfection and luciferase assays**

Total amount of DNA transfected was adjusted to 1.5 µg of DNA/35 mm dish with the empty *pSG5* and/or *pCIneo* vectors. Transfection was done the day before cells were treated with vehicle (0.1% ethanol) or 10 μM ACR. Treatment with 0.1% ethanol itself did not induce apoptosis in JHH-7 cells. Relative luciferase activity of each sample was calculated by normalization with *Renilla*-luciferase activity of the same sample.

**Gel shift assay**

Gel shift assays were performed using end-labeled oligonucleotides (consensus GC box, 5'-ATTCGATCGGGGCGGGGCGAGC-3') as described [2].

**References**

[1] Zhang J, Lesort M, Guttmann RP, Johnson GV: **Modulation of the in situ activity of tissue transglutaminase by calcium and GTP.** *J Biol Chem* 1998, **273:**2288-2295.

[2] Shimada J, Suzuki Y, Kim SJ, Wang PC, Matsumura M, Kojima S: **Transactivation via RAR/RXR-Sp1 interaction: characterization of binding between Sp1 and GC box motif.** *Mol Endocrinol* 2001, **15:**1677-1692.
