# Supplementary figures and images for: Dual induction of caspase 3- and transglutaminase-dependent apoptosis by acyclic retinoid in hepatocellular carcinoma cells
Source: Mol Cancer. 2011 Jan 9;10:4. doi: 10.1186/1476-4598-10-4 (PMC3024303; doi:10.1186/1476-4598-10-4)

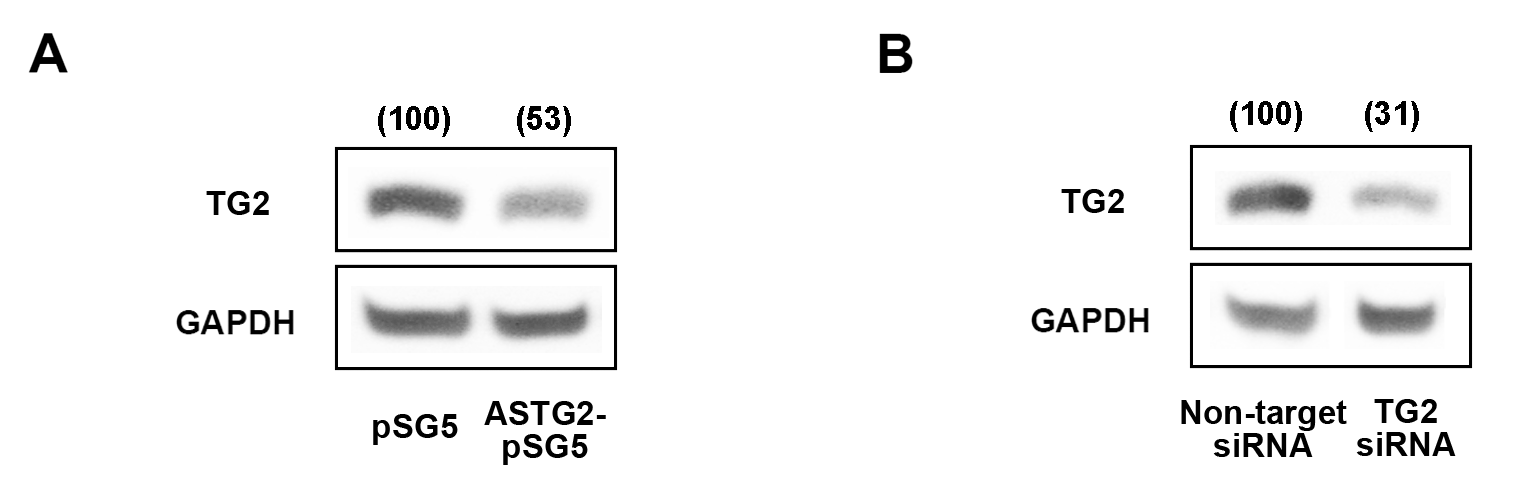

Supplement: Additional file 2 — Figure S1: Efficiency of transfection with anti-sense and siRNA to TG2 in JHH-7 cells. A, JHH-7 cells were seeded in 60 mm dishes at 6 × 10^5^/dish, and transfected with 4 μg of either empty vector (pSG5) or ASTG2-pSG5. Cells were harvested and the expression level of TG2 determined by Western blotting. Upper numbers in parentheses show the densitometrically determined relative protein abundance. B, JHH-7 cells were seeded in 60 mm dishes at 6 × 10^5^/dish, and transfected with 4 μg of vectors expressing either non-target siRNA or TG2 siRNA. Cells were harvested and the expression level of TG2 determined by Western blotting. Upper numbers in parentheses show the densitometrically determined relative protein abundance. Panels A and B show representative results from 3 different experiments with similar results. [file 1476-4598-10-4-S2.TIFF]

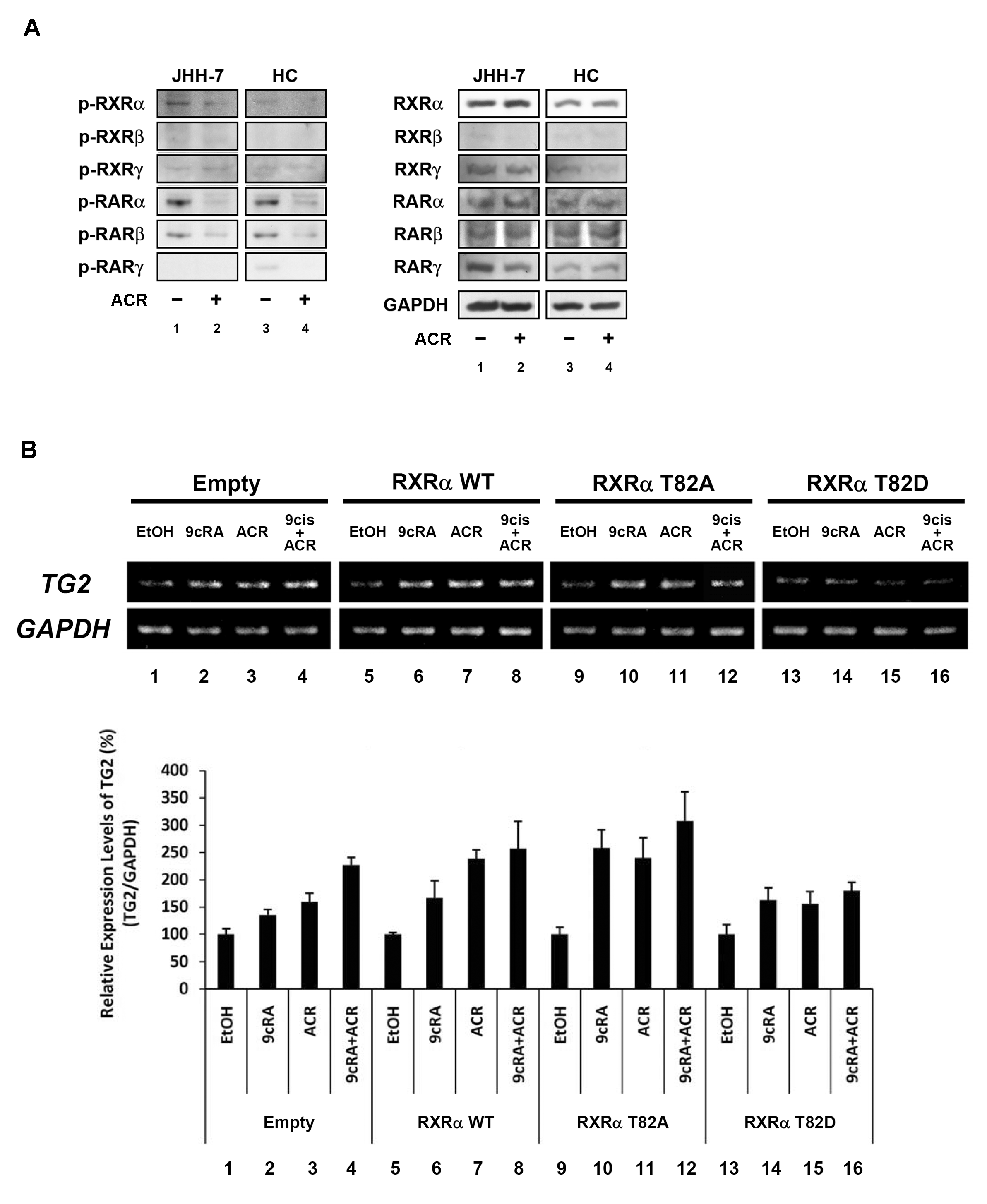

Supplement: Additional file 4 — Figure S2: ACR prevented phosphorylation and inactivation of RXRα, and stimulated the expression of TG2 in JHH-7 cells. A, JHH-7 cells (lane 1 and 2) and HC cells (lane 3 and 4) were treated with 10 μM ACR or vehicle for 12 h. Cells were harvested and nuclear extracts were prepared. Phosphoproteins affinity-purified from each nuclear extract using the Phosphoprotein Purification Kit (QIAGEN) (left panel) as well as whole nuclear extracts (right panel), were subjected to SDS-PAGE, followed by Western blotting using the indicated antibodies against 6 different RXR/RAR or GAPDH. B, JHH-7 cells were transfected with either an empty vector (columns 1-4) or vectors expressing wild-type RXRα (columns 5-8), its alanine mutant T82A (unphosphorylated form; columns 9-12), or its aspartate mutant T82 D (phosphomimic; columns 13-16). The next day cells were treated either with 9-cis RA (9cRA; 6 μM) or its vehicle, or with and/or ACR (10 μM) for 24 h. Subsequently, levels of TG2 mRNA in cell lysates were quantified by RT-PCR (upper panels) and quantitative-PCR (lower graphs), where relative expression levels of TG2 were calculated in comparison with each control and then plotted. Treatment with 1 μM 9-cis-RA also gave basically similar results (data not shown), but the data obtained under treatment with 6 μM 9-cis-RA are shown here, giving the more significant differences. Panels A and B show representative results from 3 different experiments with similar results. [file 1476-4598-10-4-S4.TIFF]

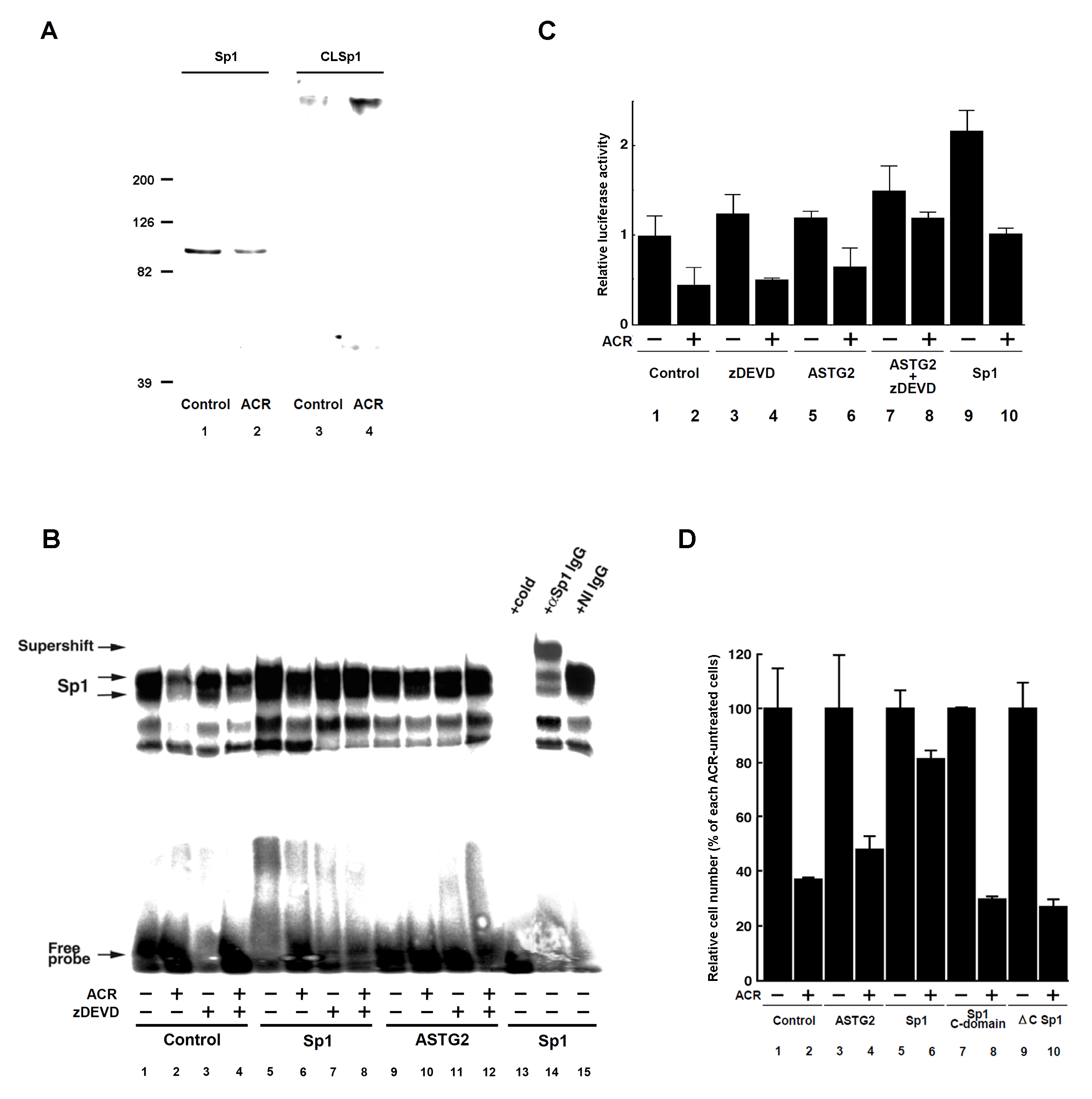

Supplement: Additional file 5 — Figure S3: Crosslinking and silencing of Sp1 in ACR-treated JHH-7 cell cultures undergoing apoptosis and its reversion by overexpression of Sp1. A, JHH-7 cells were treated with 10 μM ACR for 24 h. The cells were harvested and nuclear extracts prepared. The levels of Sp1 and CLSp1 were assessed by Western blotting with an anti-Sp1 (columns 1 and 2) and CLSp1 (columns 3 and 4) antibodies, respectively. B, JHH-7 cells were transfected with 1.5 μg of either combination of pCIneo, pSG5, Sp1-pCIneo, or anti-sense (AS) TG2-pSG5. The next day they were treated with either 10 μM ACR or its vehicle in the presence or absence of 100 μM zDEVD-fmk for 24 h. Cells were harvested and nuclear extracts prepared. Sp1 DNA-binding activity of each nuclear extract (10 μg protein) was determined by gel-shift assay, using a consensus GC box as a probe (+cold; nuclear extracts + 50-fold excess of unlabeled probe, +anti-Sp1 IgG; nuclear extracts + 2 μg of anti-Sp1 antibody, +NI IgG; nuclear extracts + 2 μg of non-immune IgG). C, JHH-7 cells were transfected with 1.5 μg of a consensus GC3-Luc reporter and Renilla-Luc, plus a combination of pCIneo, pSG5, Sp1-pCIneo or anti-sense (AS) TG2-pSG5. The next day the cells were treated with 10 μM ACR for 24 h in the presence or absence of 100 μM zDEVD-fmk. Cell lysates were prepared and luciferase activity of each cell lysate determined. Results are means ± SD (n = 3). D, JHH-7 cells were transfected with either a combination of pCIneo, pSG5, anti-sense (AS) TG2-pSG5, Sp1-pCIneo, Sp1 C domain-pCIneo, ΔC Sp1-pCIneo. The next day the cells were treated with 10 μM ACR for 24 h. The number of viable cells was determined. Results are means ± SD (n = 4). Panels A-D show representative results from 3 different experiments with similar results. [file 1476-4598-10-4-S5.TIFF]

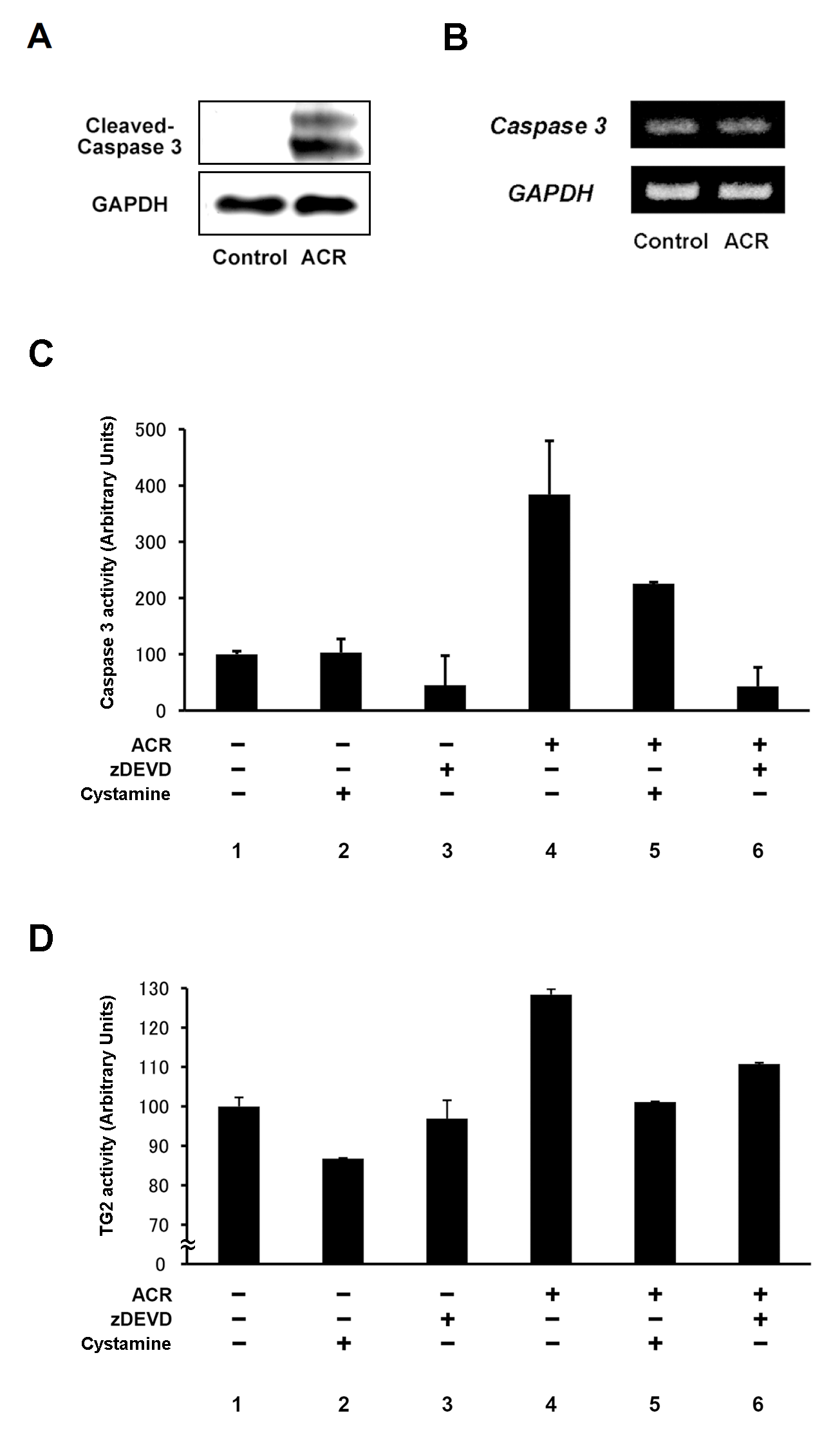

Supplement: Additional file 6 — Figure S4: ACR stimulated activation of caspase 3 and TG2 in JHH-7 cells and the crosstalk between these proteins. A and B, JHH-7 cells were treated with 10 μM ACR or the vehicle for 24 h. Cells were harvested and protein levels of activated caspase 3 and GAPDH determined by Western blots, using anti-cleaved-caspase 3 and anti-GAPDH antibodies (A); each of their mRNA expression was determined by RT-PCR (B). C, JHH-7 cells was seeded at 1 × 10^4^ cells/96 well microplates and treated with 10 μM ACR or vehicle (0.1% ethanol) for 5 h in the presence or absence of either 100 μM zDEVD-fmk or 100 μM cystamine with 0.2 mM 5-(biotinamido)-pentylamine. Caspase 3 activity was measured using a Caspase-Glo 3/7 assay kit (Promega Corp., WI) as described in attached manual. Relative caspase 3 activity of each sample was calculated by normalization with the number of viable cells in the same sample measured with a cell counting kit-8 (Dojindo; Tokyo, Japan). D, JHH-7 cells seeded in 100 mm dishes at 1.6 × 10^6^/dish were treated as in (C). TG2 activity was measured as described in Additional file 1. Relative TG2 activity of each sample was calculated by normalization with the number of viable cells in the same sample, measured with a cell counting kit-8 (Dojindo; Tokyo, Japan). Panels A-D show representative results from 3 different experiments with similar results. [file 1476-4598-10-4-S6.TIFF]
