# Supplementary material for: Dual induction of caspase 3- and transglutaminase-dependent apoptosis by acyclic retinoid in hepatocellular carcinoma cells
Source: Mol Cancer. 2011 Jan 9;10:4. doi: 10.1186/1476-4598-10-4 (PMC3024303; doi:10.1186/1476-4598-10-4)
Supplement: Additional file 3 — Table S1: Primers for RT-PCR and quantitative-PCR experiments. The list of used specific primers for RT-PCR. [file 1476-4598-10-4-S3.DOC]

**Additional file 3: Table S1.**

**Primers for RT-PCR and quantitative-PCR experiments**

| **Gene** |  | **Sequence** | **Nucleotide no.** |
| --- | --- | --- | --- |
| (RT-PCR) |  |  |  |
| Human *TG2* | Sense | AACCCCAAGTTCCTGAAG | 597-614 |
|  | Antisense | AGGTTGCTGTTCTGGTC | 916-932 |
| Human *EGFR* | Sense | CTCCGGTCAGAAAACCAAAA | 1608-1627 |
|  | Antisense | CTTCCAGACCAGGGTGTTGT | 1987-1986 |
| Human *c-Met* | Sense | TTCTGACCGAGGGAATCATC | 3558-3577 |
|  | Antisense | TTTCCAAAGCCATCCACTTC | 3928-3947 |
| Human *FGFR1* | Sense | TACCACCGACAAAGAGATGG | 1616-1635 |
|  | Antisense | CTGGCTGTGGAAGTCACTCT | 1879-1898 |
| Human *Bcl-X_L_* | Sense | TCTGGTCCCTTGCAGCTAGT | 1205-1224 |
|  | Antisense | TCAAAAGTGGCCCCTAAATG | 1567-1588 |
| Human *GAPDH* | Sense | GCAGGGGGGAGCCAAAAGGG | 395-414 |
|  | Antisense | TGCCAGCCCCAGCGTCAAAG | 942-961 |
| Human *Caspase 3* | Sense | TGGAATTGATGCGTGATGTT | 290-309 |
|  | Antisense | GGCAGGCCTGAATAATGAAA | 390-409 |
|  |  |  |  |
| ( quantitative-PCR) |  |  |  |
| Human *TG2* | Sense | TACCTGGACTCGGAAGAGGA | 445-464 |
|  | Antisense | TCTTCAGGAACTTGGGGTTG | 916-897 |
| Human *GAPDH* | Sense | CGACCACTTTGTCAAGCTCA | 972-991 |
|  | Antisense | CCCTGTTGCTGTAGCCAAAT | 1011-1030 |
