# Supplementary material for: Dual induction of caspase 3- and transglutaminase-dependent apoptosis by acyclic retinoid in hepatocellular carcinoma cells
Source: Mol Cancer. 2011 Jan 9;10:4. doi: 10.1186/1476-4598-10-4 (PMC3024303; doi:10.1186/1476-4598-10-4)
Supplement: Additional file 7 — Table S2: Suppression by ACR of metastasis and growth of human HCC cell line, JHH-7 cells transplanted into nude mice. Nude mice that had been transplanted with JHH-7 were given orally with ACR with increasing concentrations (25, 50, and 100 mg/kg/day) as described detailed in the "Methods". Serum AFP was measured. Incidence was calculated based on level of the positive-AFP (more than 6 ng/ml). Cisplatin was used as a positive control. *p < 0.05 compared to control (Dunnett's multiple comparison test), #p < 0.05 compared to control (Fisher exact test).. [file 1476-4598-10-4-S7.DOC]

**Additional file 7: Table S2.**

**Suppression by ACR of metastasis and growth of human HCC cell line, JHH-7 cells transplanted into nude mice.**

| Group | Dose | Serum AFP | AFP positive | Incidence | Number of mice |
| --- | --- | --- | --- | --- | --- |
|  | (mg/kg/day) | (ng/ml) | Mice | (%) |  |
| Control |  | 1,500 ± 73 | 13/14 | 93 | 14 |
| ACR | 25 | 532 ± 71 * | 5/12 ^#^ | 42 | 12 |
| ACR | 50 | 512 ± 75 * | 4/11 ^#^ | 36 | 11 |
| ACR | 100 | 270 ± 40 * | 3/13 ^#^ | 23 | 13 |
| Cisplatin | 5.6 | 489 ± 65 * | 4/13 ^#^ | 31 | 13 |
